# Effects of stuttering and sound avoidance on reference production and memory

**DOI:** 10.1017/S0142716425100428

**Published:** 2026-02-12

**Authors:** Si On Yoon, Morgan Schuchard, Anu Subramanian, Naomi H. Rodgers

**Affiliations:** 1 Department of Communicative Sciences and Disorders, New York University, New York, NY, USA; 2 Center for Disabilities and Development, University of Iowa Stead Family Children’s Hospital, Iowa City, USA; 3 Department of Communication Sciences and Disorders, University of Iowa, Iowa City, USA

**Keywords:** stuttering, reference, memory, dual-task

## Abstract

Adults who stutter (AWS) frequently engage in language monitoring to anticipate and manage stuttering. This linguistic monitoring may reallocate cognitive resources, with potential consequences for language production and memory. We investigated whether AWS’ increased monitoring during production imposes dual-task costs that limit encoding benefits, or whether it enhances memory through deeper conceptual engagement. Thirty-two AWS and sixty-four adults who do not stutter (AWNS) completed a referential communication task in which they described or identified pictures with an experimenter. To simulate AWS’ linguistic monitoring, half of the AWNS performed a simultaneous sound avoidance task (AWNS-SA), prohibiting certain word-initial phonemes. After the communication task, participants completed a recognition memory test for past referents. Results showed that AWS performed more similarly to AWNS than to AWNS-SA in both language production and memory, although AWS’ memory declined on a trial-by-trial basis when stuttering occurred. These findings suggest that linguistic monitoring in AWS does not impose substantial dual-task costs overall, but that stuttering moments can transiently disrupt memory encoding. Together, these results highlight the adaptive nature of linguistic monitoring in AWS and contribute to a broader understanding of how it supports language production and memory across AWS and AWNS.

## Introduction

Producing fluent speech requires the coordination of multiple cognitive processes that operate dynamically and in parallel (Levelt, 1989, 1992; Dell, 1986, 1988; Dell & Jacobs, 2016). Speakers must attend to and perceive referents in their discourse context, retrieve lexical and phonological information, and flexibly update memory representations to ensure appropriate reference across discourse contexts (e.g., “the coffee,” “the hot coffee,” “the Cappuccino,” “it”). At the same time, they must monitor their speech output to detect and correct phonological or semantic errors before or during articulation (Levelt, 1983, 1989; Postma, 2000). The present study investigates how the demands of this monitoring process affect cognitive resource allocation during language production in adults who stutter (AWS). Specifically, we tested whether AWS allocate cognitive resources differently from adults who do not stutter (AWNS) and how such allocation influences subsequent memory encoding and retrieval.

### Stuttering and language production

#### Stuttering

Stuttering is a multifaceted neurodevelopmental condition that involves behavioral, cognitive, and social-emotional components that interact dynamically as they navigate their daily lives—from speech production to life decisions. Behaviorally, stuttering results from disruptions in speech motor planning that lead to breaks in the forward flow of linguistic output (Chang et al., 2019). Stuttering-like disfluencies include sound/syllable repetitions (e.g., “muh-muh-muh-my”), sound prolongations (e.g., “mmmmy”), and blocks in which there is a stoppage of airflow and movement before releasing the sound (e.g., “---[tension]---my”). Moments of stuttering can also involve associated behaviors like closing one’s eyes, craning one’s head, moving one’s limbs, or exerting excess physical tension—behaviors that people who stutter may consciously or subconsciously do to try to escape moments of stuttering.

Cognitively, the experience of stuttering is not limited to these surface-level behaviors; rather, it involves complex internal processes of monitoring and anticipation that influence how speakers plan and produce language. It is common for AWS to anticipate moments of upcoming stuttering—an interoceptive awareness that develops with age (Jackson et al., 2018). This anticipation requires linguistic monitoring and can place cognitive demands on the speaker as they make momentary decisions about how to navigate potential trigger words. Such heightened linguistic monitoring may impose additional demands on attentional cognitive resources, potentially disrupting language production processes compared to AWNS.

Socially and emotionally, repeated experiences of stuttering and ensuing listener reactions can evoke feelings of anxiety and embarrassment, which may further heighten self-focused attention and exacerbate speech monitoring demands (Iverach & Rapee, 2014). These emotional reactions can become more pronounced in social contexts where fear of negative evaluation or social anxiety is heightened (Bauerly, 2024).

Over time, the interplay among these behavioral, cognitive, and social-emotional factors can give rise to a range of strategic avoidance behaviors to minimize overt stuttering-like disfluency, such as by word substitutions and circumlocutions (Jackson et al., 2019). While these avoidances can reduce stuttering that listeners hear, they may also increase the speaker’s cognitive load and change the allocation of cognitive resources during language planning. Understanding how such monitoring and avoidance processes shape language production and downstream memory encoding among AWS is central to the present study.

#### Language production in AWS

Language production is a multistage process involving conceptualization, formulation, and articulation of messages (Levelt, 1989, 1992; Dell, 1986, 1988; Roelofs, 1992, 1997). Speakers first conceptualize their intended message, then select lexical items and organize them syntactically, and finally articulate the utterance while monitoring for errors via both inner and outer speech loops (Levelt, 1983, 1989; Postma, 2000). These processes rely on domain-general cognitive resources, including cognitive control to maintain focus (Strijkers et al., 2011), monitoring to detect and prevent errors (Nozari & Novick, 2017), working memory to activate lexical and phonological representations (Schwering & MacDonald, 2020), and attentional selection of appropriate linguistic units (Ferreira & Pashler, 2002).

Research suggests that language production mechanisms are disrupted in AWS at both the lexical-semantic and phonological levels (Dell & O’Seaghdha, 1992; Maxfield, 2015; Maxfield et al., 2010, 2012, 2015). While stuttering is typically associated with disruptions in motor speech execution (Smith & Weber, 2017), AWS also exhibit differences in lexical access and production efficiency. For example, in picture-naming tasks, AWS produce naming errors more frequently and demonstrate slower retrieval of low-frequency words than AWNS (Newman & Ratner, 2007). One prominent hypothesis is that AWS allocate cognitive resources differently than AWNS during language production, engaging in increased linguistic monitoring that diverts attention from primary production processes. Support for this hypothesis comes from electrophysiological studies showing an attenuated P3 component in AWS during language tasks involving concurrent cognitive demands (Maxfield et al., 2016). The P3 component reflects the availability of cognitive resources for target processing (Luck, 1998), and its attenuation in AWS suggests that linguistic monitoring imposes a cognitive load. Importantly, AWS and AWNS exhibit comparable P3 magnitudes in non-linguistic tasks, further supporting the notion that AWS experience increased cognitive demands specifically during linguistic-related activities.

The consequences of heightened linguistic monitoring in AWS—which may function as a dual task, requiring speakers to produce speech while simultaneously monitoring for stuttering-like disfluencies—extend beyond fluency to broader cognitive functions. If AWS engage in such dual-task activities, their ability to encode linguistic information for later retrieval may be impaired. This raises the possibility that AWS’ linguistic monitoring affects not only their real-time speech fluency but also their memory representations of discourse. Alternatively, monitoring may not always be detrimental; increased self-monitoring could also deepen engagement with the linguistic material, fostering more distinctive or elaborated representations that enhance memory (McCurdy et al., 2020; Yoon et al., 2016). The present study tests these possibilities by examining how language production and monitoring demands influence memory encoding in AWS.

### The interface between language and memory in AWNS

#### Language production and modifier use

**Figure 1.**
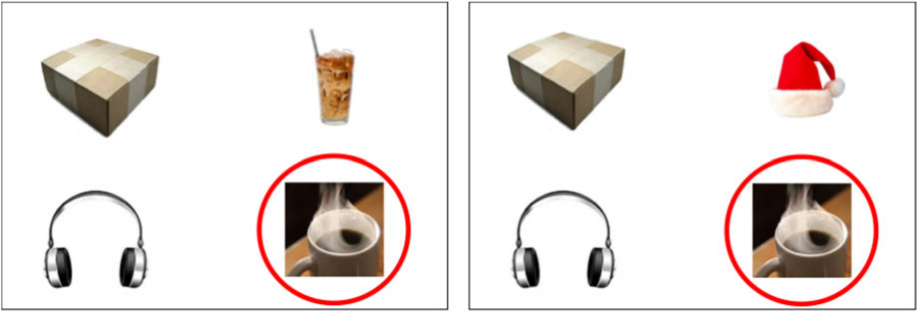
Example stimuli in the contrast (left) and non-contrast condition (right) for speakers.

When cognitive resources are taxed, such as under high attentional load or divided focus, speakers often exhibit increased error rates, slower lexical access, and reduced fluency (Ferreira & Pashler, 2002; Schnur et al., 2009). These demands are evidenced during referential communication, in which speakers must select expressions that effectively identify intended referents for listeners. In such contexts, speakers use modifiers (e.g., “the hot coffee” in the presence of hot and iced coffee; see Figure 1, left panel) to disambiguate referents. While modifiers serve an essential communicative function, speakers sometimes overproduce them even when no contrasting alternative exists (e.g., “the hot coffee” when no contrast exists) (Pechmann, 1989; Tarenskeen, Broersma, & Geurts, 2015).

Overmodification has been linked to multiple cognitive and communicative factors. It may arise from limited attentional control or increased processing load, leading speakers to rely on redundant features (Bannon, 2019). Alternatively, speakers may deliberately overspecify to ensure listeners’ comprehension, particularly when they anticipate potential misunderstandings (Rubio-Fernandez, 2016; see also Yoon, 2018). Since language production relies on domain-general control mechanisms to manage competition among lexical alternatives (Nozari, 2018), disruptions in attentional allocation—such as those possibly induced by increased linguistic monitoring among AWS—may influence modifier use and, in turn, potentially affect referential specificity.

#### The interaction between memory and language

Language production fundamentally shapes how individuals form and retain memory representations. In conversation, speakers typically remember referents they describe better than listeners who hear them (Yoon, Benjamin, & Brown-Schmidt, 2016, 2021; McKinley, Benjamin, & Brown-Schmidt, 2017; Zormpa et al., 2019). This “speaking benefit” aligns with the well-established *generation effect* in memory research (Slamecka & Graf, 1978), wherein self-generated information is encoded and retrieved better than passively received input. Producing language requires speakers to select lexical items, plan syntactic structures, and integrate conceptual and linguistic information, all of which promote deeper, more elaborative encoding.

Moreover, when speakers describe referents with greater linguistic specificity—such as by adding modifiers—they show enhanced memory for those referents (Yoon et al., 2016; 2021; Yoon & Brown-Schmidt, 2023). For example, a speaker who says *“*the hot coffee*”* instead of simply *“*the coffee*”* must retrieve and integrate visual, conceptual, and linguistic information to distinguish that object from others in the same context. Similarly, producing a phrase like *“*the hot coffee in the gray mug*”* requires selecting features that uniquely identify the intended referent and engaging additional cognitive resources during message formulation. The extra information—typically expressed through modifiers—also shapes which aspects of the referent are encoded in memory representations. As a result, increased linguistic and conceptual elaboration strengthens the memory trace for both the referent and its associated features (Broadshaw & Anderson, 1982; Yoon et al., 2016). Thus, generating more specific or contrastive descriptions promotes deeper encoding, which may explain why speakers typically remember the items they describe more accurately than listeners who hear them.

Among AWS, however, this language-memory interaction may differ. Heightened linguistic monitoring during language production may divert cognitive resources from encoding referential content, potentially reducing the typical memory advantage observed in AWNS. Conversely, such monitoring may deepen processing by increasing engagement with linguistic planning. The present study examines how monitoring during language production influences memory in AWS relative to AWNS, providing new insight into how cognitive control and fluency interact in communication.

### The current research

Although AWS are known to often anticipate and attempt to avoid stuttering, little is known about how these behaviors influence cognitive resource allocation during language production and subsequent memory encoding. In the present study, we applied a psycholinguistic paradigm to test how increased linguistic monitoring in AWS affects both language production and memory for discourse referents.

We hypothesized that heightened monitoring may serve as a dual task, requiring AWS to divide attention between linguistic formulation and monitoring for potential stuttering. This divided attention could disrupt efficient linguistic formulation and reduce the depth of encoding for referential information, leading to poorer memory performance. Alternatively, increased monitoring may promote deeper engagement with linguistic and conceptual content, counteracting potential dual-task costs and resulting in memory performance comparable or superior to AWNS.

To test these possibilities, we compared AWS with AWNS in a referential communication task designed to elicit modifier use. A subgroup of AWNS engaged in a secondary sound-avoidance condition (AWNS-SA), simulating the attentional load AWS may experience during speech monitoring. We predicted that if monitoring primarily taxes cognitive resources, AWS performance would resemble that of the AWNS-SA group, showing differences in modifier use (e.g., omitting necessary or producing unnecessary modifiers) and reduced memory for referents. However, if monitoring instead enhances engagement and processing, AWS may demonstrate preserved memory representations relative to AWNS. By testing these competing hypotheses, this study aims to provide insight into how linguistic monitoring in AWS affects both real-time language production and memory formation and retrieval, shedding light on the cognitive mechanisms underlying stuttering and its broader cognitive consequences.

## Experiment method

The Institutional Review Board at the University of Iowa approved this study. All data are publicly available on the Open Science Framework (OSF) and can be accessed at https://osf.io/xa76n/overview.

### Participants

A total of 96 native English speakers (ages 18–45) participated in the study, receiving $10 per hour for their participation. Inclusion criteria required that participants (1) report English as their native language and (2) self-report normal hearing and visual acuity (corrected or uncorrected). Sixty-four AWNS participants with no history of communication disorders were recruited from the community. Additionally, 32 AWS participants were recruited from the last author’s research registry, which includes individuals who stutter and have previously participated in her research, consenting to be contacted for future studies. All AWS participants self-identified as people who stutter, reported childhood onset of stuttering, and indicated no co-occurring speech, language, or literacy issues.

Each of the three groups—AWS, AWNS, and AWNS-SA—had an equal number of participants. The AWS group (*n* = 32; *M*_*age*_ = 29.31 years, *SD* = 7.25) included 14 women, 17 men, and one non-binary person. The AWNS group (*n* = 32; *M*_*age*_ = 28.38 years, *SD* = 8.72) included 23 women and 9 men. The AWNS-SA group (*n* = 32; *M*_*age*_ = 28.59 years, *SD* = 8.22) included 13 women, 18 men, and one non-binary person. The AWNS-SA group was designed to simulate the divided attentional demands that AWS may experience in language production due to sound avoidance.

The groups were matched on age and education level, with no significant between-group differences in age (*F*(2,93) = 0.12, *p* = .89) or education level (*F*(2,92) = 1.31, *p* = .28). Participant demographics are provided in Table 1.

**Table 1.** Demographic information (mean and standard deviation) for each group

	AWS (*n* = 32)	AWNS (*n* = 32)	AWNS-SA (*n* = 32)
Age in years	29.31 (7.25)	28.38 (8.72)	28.59 (8.22)
Education in years	16.19 (1.66)	15.06 (2.21)	16.06 (1.79)

*Note:* One participant in the AWS group did not report their education level.

### Procedure

Following the consent process using REDCap electronic data capture tools hosted at the University of Iowa (Harris et al., 2009; Harris et al., 2019), participants completed the referential communication task, a series of cognitive measures, and an unexpected recognition memory test. To prevent ceiling effects in memory performance (Shepard, 1967), a filler task^1^ was introduced before the recognition memory test (see also Yoon et al., 2016, 2021; Saryazdi, Nuque, & Chambers, 2022). The study was conducted via Zoom and lasted approximately one hour. Audio and video recordings were collected for data coding.

#### Referential communication task

At the start of the scheduled session, the experimenter emailed participants PowerPoint slides containing the stimuli for the referential communication task. Participants and the experimenter were assigned to begin as either the speaker or the listener. To simulate a face-to-face conversation, Zoom was set to speaker view (self-view off), ensuring that participants could see only the experimenter’s face and vice versa.

**Figure 2.**
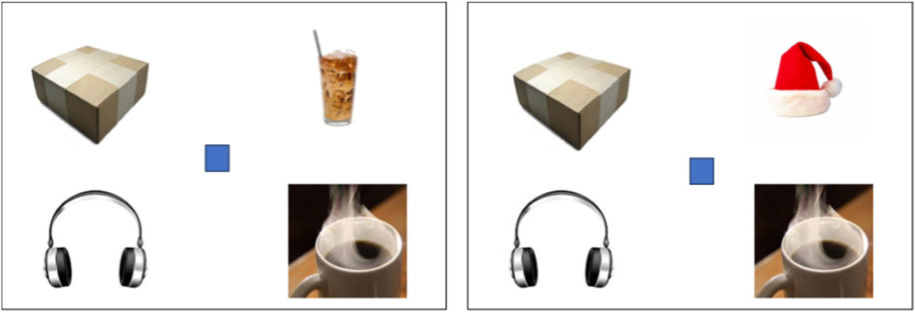
Example stimuli in the contrast (left) and non-contrast condition (right) for listeners. Listeners used their mouse to move the blue square to the target item once the speaker provided the verbal description.

For each trial, both participants viewed a slide containing four images (Figures 1 and 2). The target image was marked with a red circle on the speaker’s slide only (Figure 1). The speaker described the target image to the listener (e.g., *“The target is coffee”*). While the carrier phrase (*“The target is …”*) was not required, participants often naturally used full sentences (e.g., *“I see …”* or *“The picture is …”*). The listener then used their mouse to move a blue square onto the target image (Figure 2). This interactive task mimicked natural communication in that speakers could describe the image freely, but they were not allowed to use location-based descriptions (e.g., *“The bottom right one”*). Listeners could ask for clarification if a description was insufficiently specific (e.g., *“coffee”* when both hot and iced coffee were present).

Once the listener selected the target, they instructed the speaker to proceed to the next trial. Each participant completed 48 trials in their initial role before taking a short break and switching roles. The order of speaking and listening was counterbalanced across participants.

In total, participants completed 96 trials, consisting of 48 critical trials (24 production + 24 comprehension) and 48 filler trials (24 production + 24 comprehension). In critical trials, the discourse context was manipulated to include either a contrast item or no contrast item (Figures 1 and 2). In the contrast condition (12 production + 12 comprehension trials), a semantically related contrast image (e.g., target = hot coffee, contrast = iced coffee) was present, requiring a modified expression (e.g., *“the hot coffee”*) to identify the target. In the non-contrast condition (12 production + 12 comprehension trials), the target image had no related items (e.g., *“the coffee”* was sufficient). The target and contrast items were counterbalanced across eight lists (e.g., the hot coffee was the target in one list and a competitor in another). Additionally, 48 filler trials were included to increase stimulus variability. Half of these trials required a modifier, while the other half did not. The same filler trials were used across lists.

##### Sound avoidance condition (AWNS-SA group)

Participants in the attention-divided group (AWNS-SA) simultaneously performed a sound avoidance task during the referential communication task. This task was adapted from the word taboo paradigm used to elicit aphasia-like behaviors in neurotypical adults (Meffert et al., 2011). Participants were instructed to avoid using words that began with four common phonemes: /f/, /k/, /p/, and /s/. These phonemes were selected as they were the second, third, fourth, and fifth most common word-initial sounds in the target items. For example, the word *“coffee”* (initial /k/) was prohibited, requiring participants to use an alternative word (e.g., *“espresso”* or *“drink”*). On 38% of trials, the target item’s name began with a prohibited phoneme. Participants were not provided specific alternative strategies beyond selecting different nouns.

#### Unexpected recognition memory test

After completing the referential communication task and the cognitive measures, participants took an unexpected recognition memory test. In each trial, they were shown a single image and asked whether they had seen it during the previous communication task (e.g., “Have you seen this picture before?”). Participants clicked “yes” if they recognized the image from the communication task, which could include target images (e.g., the hot coffee in Figure 1), contrast images (e.g., the iced coffee in Figure 1), and filler images (e.g., the key from a filler trial). They clicked “no” if they did not recall seeing the image during the task.

Half of the pictures were previously seen (“old” items), and the other half were new, previously unseen images. The old items included 48 critical target images (e.g., the hot coffee) that had been described by either the participant or the experimenter, 48 contrast images (e.g., the iced coffee), which were present but not described during the communication task, and 48 filler images (e.g., the key from a filler trial), half of which had been described, while the other half had been presented but not described. Additionally, there were 144 new images from the same categories as the old items (e.g., a different image of hot coffee). In total, the memory test consisted of 288 trials assessing participants’ recognition of past referents.

#### Stuttering anticipation scale (AWS only)

At the end of the study, AWS participants rated their anticipated stuttering and avoidance behaviors during the referential communication task on a 5-point Likert scale^2^ (*1* = Never, *5* = Always). On average, AWS rated their anticipation at 3.07 (*SD* = 1.27, range = 1–5), indicating they sometimes expected to stutter. Their avoidance behavior ratings averaged 2.41 (*SD* = 1.37, range = 2–5), indicating they sometimes avoided certain words. These patterns align with prior findings on individual differences in stuttering anticipation and avoidance (Jackson et al., 2018; Rodgers & Jackson, 2021).

Thirty AWS also completed the *Stuttering Anticipation Scale* (SAS; Jackson et al., 2018). On this measure, AWS first rated how often they anticipated stuttering in their daily lives on a 5-point Likert scale (*M* = 3.80, *SD* = 0.81, *range* = 2–5). Using the same Likert scale, they then rated how frequently they engaged in 25 common behavioral responses to anticipation. The average sum score was 66.03 (*SD* = 10.41, *range* = 43–91) out of a possible range of 25–125.

### Coding, planned analyses, and predictions

#### Communication task

We transcribed the audio recordings from the communication task and coded whether participants produced a modifier in each trial when they spoke. The analysis focused on the initial referring expression produced by the speaker before receiving any feedback from the experimenter, minimizing the potential influence of partner feedback on participants’ descriptions (see Yoon et al., 2017). Specifically, we examined the use of pre-noun modifiers (e.g., “the hot coffee”), which reflect pre-planned language prior to production (Brown-Schmidt & Konopka, 2008). Post-noun modifiers (e.g., “the coffee that is hot”) were excluded because it is less clear whether they result solely from the speaker’s internal language planning processes or are influenced by partner feedback, including both verbal and backchannel cues. For instance, a partner’s hedging may encourage speakers to produce longer utterances, potentially leading to the addition of post-noun modifiers.

We also coded whether AWS stuttered in each trial using a binary coding system: fluent (coded as 0) or stuttered (coded as 1). If a participant produced any stuttering-like disfluencies (i.e., sound/syllable repetitions, prolongations, blocks) at any point while describing the target image—regardless of the type or frequency of stuttering, or whether the stutter occurred on the target noun (e.g., “coffee”) or another word in the utterance—it was coded as 1. Fillers (e.g., “um,” “uh”) were not coded as stuttering, as it is often unreliable to determine whether fillers reflect stuttering avoidance or typical non-stuttered disfluencies due to language processing stalls^3^.

The final dataset comprised 2,264 picture descriptions (24 critical production trials per participant), excluding 40 trials in which participants did not produce a noun (e.g., producing only a modifier). All of these excluded trials came from the AWNS-SA group.

To analyze the modifier data, we used a binomial (logit-link) mixed-effects model with contrast type (contrast vs. non-contrast) and Group (dummy-coded, with AWS as the reference group: (1) AWS vs. AWNS and (2) AWS vs. AWNS-SA) as fixed effects, with participants and items as random intercepts. Random slopes were included whenever possible. The dependent variable was binary—whether the target noun was modified or not.

All models were fit using the lme4 package (Bates et al., 2015) in R (version 4.1.0; R Core Team, 2021), using the maximal random effects structure for participants and items. The R package “buildmer” was used to identify a convergent maximal random effects structure (see Voeten, 2023).

#### Unexpected recognition memory test

Following the analysis process in Yoon et al. (2016, 2021), we calculated discriminability (*d′*) in the unexpected recognition memory test to assess participants’ ability to distinguish between old and new items. Discriminability was determined by subtracting the standardized false alarm rate (e.g., a “yes” response to a new item they had not seen before, such as a different hot coffee) from the standardized hit rate (e.g., a “yes” response to an old item they had seen before, such as the hot coffee in Figure 1). A higher *d’* value indicates better memory of the target item.

Inferential statistics for the recognition memory data were conducted for target items using a logistic mixed-effects model. The model included contrast type (contrast vs. non-contrast), group (dummy-coded with AWS as the reference; (1) AWS vs. AWNS and (2) AWS vs. AWNS-SA), role (speaker vs. listener), and actual item type (old vs. new) as fixed effects, with participants and items as random intercepts. Random slopes were also included. The dependent measure was binary, indicating whether the participant responded “yes” (old image = 1) or “no” (new image = 0).

The intercept term in the model measured response bias, specifically whether participants showed a general inclination to respond “yes” or “no.” Actual item type (old vs. new) was examined to determine whether participants were more likely to respond “yes” when the item was old, representing the primary measure of memory (see Fraundorf, Watson, & Benjamin, 2010; Wright, Horry, & Skagerberg, 2009). The beta weights associated with the fixed effects indicated the influence of these variables on response bias. Finally, the *interaction* between contrast type, role, group, and actual item type was analyzed to assess how these factors affected memory for past items.

The final dataset for the unexpected recognition memory test included 9,024 target items (48 old and 48 new items per participant), excluding two AWS participants due to experimenter errors.

We also examined the interaction between stuttering during language production and memory. As part of these exploratory analyses, we investigated whether stuttering during a trial in the communication task or SAS scores affected memory for past referents.

### Hypotheses

Our primary goals were to examine (1) modifier use in the communication task, (2) memory performance, and (3) the relationship between referential expressions produced during the communication task and the accuracy of subsequent memory performance.

In the referential communication task, we hypothesized that attentional disruptions (e.g., those associated with anticipating or attempting to avoid moments of stuttering) would affect the production of appropriate referring expressions. Specifically, if increased monitoring acts as a dual-task burden that draws resources away from linguistic formulation, speakers in the AWS and AWNS-SA groups would produce fewer modified expressions in the contrast condition compared to speakers in the AWNS group. Alternatively, if linguistic monitoring promotes deeper engagement with conceptual and linguistic processing, AWS may show preserved or even enhanced modifier use, similar to that of the AWNS group.

With respect to memory, we hypothesized that AWS and AWNS-SA would exhibit comparable memory performance, both showing reduced memory accuracy relative to AWNS, reflecting the shared attentional demands of monitoring or dual-task performance. However, if monitoring enhances rather than depletes cognitive resources, facilitating more elaborative encoding, AWS may demonstrate equivalent or better memory performance than AWNS.

Finally, among AWS, if overt stuttering or behavioral reactions to stuttering anticipation impose additional cognitive costs, we hypothesized that stuttering moments would be associated with reduced memory accuracy within the AWS group. Together, these hypotheses allowed us to test whether monitoring during language production is primarily a resource-dividing cost or an engagement-driven mechanism that may support memory encoding.

## Results

### Results in the referential communication task

**Figure 3.**
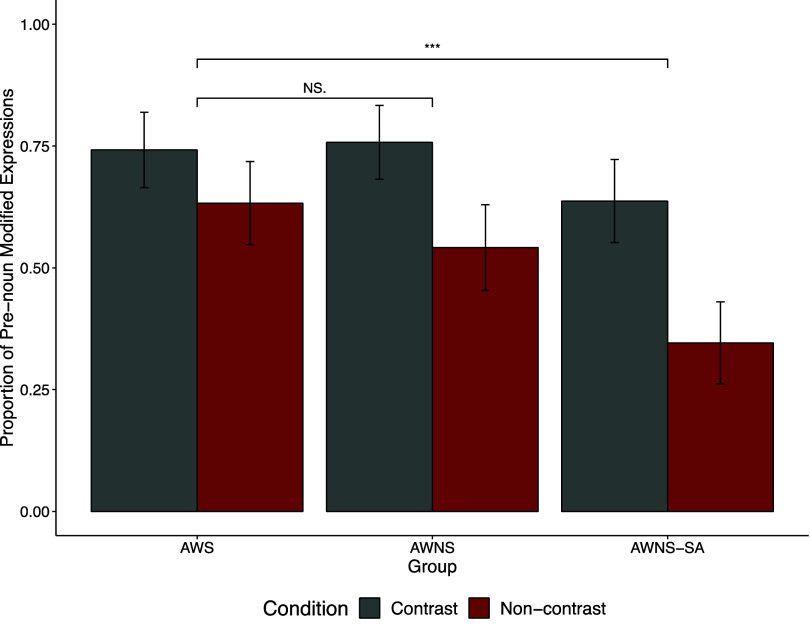
Proportion of pre-noun modified expressions in the referential communication task.

The results of the communication task (Figure 3, Table 2) indicate that speakers across all three groups used more pronoun modifiers to describe target images in the contrast condition (e.g., *hot coffee*) than in the non-contrast condition (e.g., *coffee*).

**Table 2.** Proportion of modified expressions in the referential communication task

	AWS	AWNS	AWNS-SA
Contrast condition	74.22%	75.78%	59.90%
Non-contrast condition	63.28%	54.17%	33.07%

We analyzed the use of modified expressions using a binomial (logit-link) mixed-effects model (Table S2). The model revealed significant main effects of contrast (contrast vs. non-contrast; *b* = −0.58, *z* = −3.14, *p* < .01) and Group2 (AWS vs. AWNS-SA; *b* = −1.05, *z* = −4.66, *p* < .0001). Participants in all three groups produced more modifiers in the contrast condition than in the non-contrast condition. Additionally, AWS participants produced more modifiers overall than AWNS-SA participants. However, the overall modifier rate did not significantly differ between AWS and AWNS participants (*b* = −0.19, *z* = −0.85, *p* = .39).

The significant interaction between Group 1 and contrast (*b* = −0.56, *z* = −2.30, *p* = .02) was driven by a larger contrast effect (i.e., the difference in pre-noun modifier rates between the contrast and non-contrast conditions) in the AWNS group (*b* = −1.15, *z* = −5.84, *p* < .0001) compared to the AWS group (*b* = −0.56, *z* = −3.34, *p* = .001).

Similarly, the significant interaction between Group 2 and contrast (*b* = −0.86, *z* = −3.53, *p* < .001) was driven by a larger contrast effect in the AWNS-SA group (*b* = −1.38, *z* = −7.50, *p* < .0001) than in the AWS group (*b* = −0.56, *z* = −3.34, *p* = .001). Interestingly, the AWS group exhibited a smaller contrast effect than the other two groups. This difference was due to a higher rate of modifiers in the non-contrast condition; AWS participants produced more unnecessary modifiers even when they were not required in the local context, such as when only one coffee was present on the right panel in Figure 1.

A separate mixed-effects model comparing only the AWNS and AWNS-SA groups revealed significant main effects of Group (AWNS vs. AWNS-SA; *b* = −0.88, *z* = −3.68, *p* < .001) and contrast (*b* = −1.29, *z* = −7.90, *p* < .001). However, the interaction between Group and contrast was not significant (*b* = −0.38, *z* = −1.28, *p* = .20).

### Results in the unexpected recognition memory test

**Figure 4.**
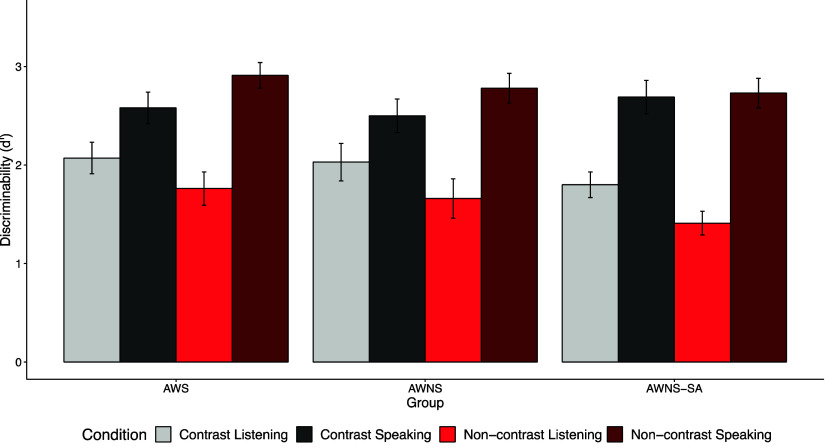
Discriminability (*d′*) for the target items in the unexpected recognition memory test.

We analyzed recognition memory data for the target (Figure 4), following the methods in Yoon et al. (2016, 2021). Recognition memory for target items was assessed using a binomial (logit-link) mixed-effects model (Table S3 in the appendix). The model included group (dummy-coded with AWS as the reference; (1) AWS vs. AWNS and (2) AWS vs. AWNS-SA), role (speaker vs. listener), and actual item type (old vs. new) as fixed effects^4^. The interactions between actual item type and fixed effects are examined, controlling response bias for “yes.” Across all groups, participants exhibited better memory for past referents when they described the item themselves rather than when they listened to the picture description (*b* = 1.10, *z* = 4.88, *p* < .001), consistent with the generation effect (Slamecka & Graf, 1978). Interestingly, the AWS group showed better memory for past referents than the AWNS-SA group (*b* = −0.53, *z* = −3.49, *p* < .01) across both speaker and listener roles. However, memory performance did not differ significantly between the AWS and AWNS groups (*b* = −0.13, *z* = −0.82, *p* = .41). These findings suggest that AWS formed memory representations as robust as those of AWNS participants, with speaker versus listener roles influencing memory representations across all groups.

A separate mixed-effects model comparing only the AWNS and AWNS-SA groups revealed significant effects of Group (AWNS vs. AWNS-SA; *b* = −0.63, *z* = −2.01, *p* = .04), Role (speaking vs. listening; *b* = 1.21, *z* = 4.74, *p* < .001), and contrast (*b* = 0.45, *z* = 2.02, *p* = .04).

### Effect of stuttering on memory

We further examined how stuttered trials influenced AWS participants’ memory for target images. Specifically, we investigated whether their memory performance differed between fluent and stuttered trials. During the communication task, AWS stuttered in 23% of trials (25.6% in the contrast condition, 20.8% in the non-contrast condition)^5^. As shown in Table 3, in the non-contrast condition, AWS’ memory for target items declined when they stuttered compared to when they did not. However, in the contrast condition, stuttering did not significantly impact memory performance.

**Table 3.** Accuracy of the memory test as a function of stuttering in the corresponding trial in the referential communication in AWS

	Fluent trials	Stuttered trials
Contrast	94.0%	93.5%
Non-contrast	93.7%	84.0%

AWS’ memory for target items and the occurrence of stuttering were analyzed using a binomial (logit-link) mixed-effects model, with contrast (contrast vs. non-contrast) and stuttering (whether the participant stuttered in that trial) as fixed effects. The dependent measure was whether the participant correctly recognized the target item. The model revealed significant main effects of contrast (*b* = −7.18, *z* = −3.73, *p* < .001) and Stuttering (*b* = −1.40, *z* = −2.18, *p* = .03). Trial-by-trial analysis indicated that AWS exhibited better memory for target items in the contrast condition than in the non-contrast condition, likely due to the higher modification rate in the contrast condition. Additionally, memory performance for target items declined when AWS stuttered while describing the item. However, the interaction between Condition and Stuttering was not significant (*b* = 0.44, *z* = 0.36, *p* = .72).

We also examined whether SAS responses (i.e., AWS participants’ general stuttering anticipation and behavioral reactions to anticipation) were associated with performance in the communication task and memory test. Neither the overall degree of stuttering anticipation nor the degree of specific behavioral reactions to anticipation was significantly associated with language production (*b* = 0.03, *z* = 0.15, *p* = .88; *b* = 0.02, *z* = 1.40, *p* = .16) or subsequent memory performance (*b* = −0.45, *z* = −1.67, *p* = .10; *b* = −0.02, *z* = −1.20, *p* = .23).

## Discussion

In this study, we investigated how linguistic monitoring in AWS influences both language production and memory for discourse referents. Specifically, we were interested in how stuttering anticipation and potential stuttering avoidance shape the allocation of cognitive resources during language production and how these processes influence subsequent memory for previously mentioned referents. During the referential communication task, participants either described target images to a partner or listened to their partner’s descriptions. Afterward, their memory for past referents was tested. To examine the effect of attentional demands associated with monitoring and avoidance behaviors, we included a comparison group of AWNS and a subgroup of AWNS who performed a simultaneous sound avoidance task (AWNS-SA). Below, we discuss three primary findings:

1. Sensitivity to discourse context and increased modifier use in AWS: participants across all groups adjusted their language production according to discourse context, producing more modifiers when a semantically related contrast item was present compared to when it was absent. Compared to the other two groups, AWS produced more unnecessary modifiers when the discourse context did not require them.
2. Memory performance across groups: AWNS-SA exhibited reduced memory performance compared to AWS and AWNS, suggesting that explicit divided attention required for the sound avoidance task impaired memory encoding in AWNS-SA, whereas AWS and AWNS showed comparable memory performance.
3. Effect of stuttering on memory: trial-level analyses revealed that stuttering events disrupted memory for target items in AWS.

### Sensitivity to discourse context in language production

Across all three groups, speakers produced more modifiers in contrast trials than in non-contrast trials, demonstrating sensitivity to discourse context even when performing a secondary task (AWNS-SA and possibly AWS). However, AWNS-SA produced fewer modifiers overall, consistent with the idea that divided attention limits resources available for message formulation. Their efforts to avoid taboo sounds in the simultaneous sound avoidance task may have made it harder to retrieve appropriate words quickly. Alternatively, their reduced modifier use may reflect both lexical retrieval difficulties and cognitive interference resulting from maintaining the sound-avoidance constraints.

Interestingly, AWS and AWNS-SA differed in their use of modifiers. AWS produced more modifiers than AWNS-SA, suggesting that the cost of monitoring differs from that of performing an unfamiliar secondary task. Unlike the AWNS-SA group, AWS may be more habituated to monitoring and redistributing attentional resources during language production, minimizing its disruptive effects on language production. Rather than reflecting a simple resource deficit, these findings suggest that monitoring in AWS may be better conceptualized as a dynamic control process—one that enhances engagement with language formulation.

AWS produced more modifiers than both AWNS and AWNS-SA, particularly in the non-contrast condition where modifiers were not necessary. One possible explanation is that AWS uses modifiers as a compensatory strategy to ensure their partner fully understands their intended message. Another possibility is that this reflects overcompensation, in which they provide more words than needed even when those extra details do not improve communication. This overproduction may stem from self-blame or perceived guilt about stuttering, as suggested by Boyle (2020), with speakers attempting to show speakers their fluency by overelaborating.

### Memory performance across groups

The memory findings contribute to understanding of the speaking benefit in memory—the advantage of producing over hearing words—operates under varying cognitive demand. Specifically, they highlight the differences between speakers engaged in typical conversation (AWNS) and those performing under a divided attention condition (AWS and AWNS-SA). Consistent with the generation effect (Slamecka & Graf, 1978), results showed that producing words rather than passively hearing them enhanced memory—even in contexts that taxed attentional resources.

However, AWNS-SA exhibited reduced memory accuracy compared to both AWS and AWNS, suggesting that explicit divided attention during language production hinders referential encoding. In contrast, AWS’ memory performance was comparable to that of the AWNS, despite their ongoing monitoring demands. This finding indicates that linguistic monitoring in AWS does not uniformly tax cognitive resources and instead supports the hypothesis that increased monitoring may, under certain conditions, deepen engagement with linguistic processing, thereby offsetting potential dual-task costs.

Although prior work suggests that modified expressions enhance memory for referents (Yoon et al., 2016), AWS’ higher modifier rate in the referential communication task did not yield better memory performance. This finding appears to suggest that increased linguistic monitoring can reallocate cognitive resources in ways that limit encoding benefits. However, it may instead reflect that monitoring promotes more detailed—but not necessarily durable—encoding. Thus, monitoring does not always impair memory; its effects likely depend on which control resources are engaged and the balance between effortful monitoring and meaningful engagement (Nozari & Novick, 2017).

Why, then, did AWNS-SA demonstrate lower memory performance than AWS? If we assume that both groups engaged in sound avoidance, one might have expected comparable performance between them. However, our findings suggest that linguistic monitoring in AWS does not uniformly tax cognitive resources. For AWS, linguistic monitoring is a habitual process deeply integrated into speech planning, whereas for AWNS-SA, the sound-avoidance task introduced a novel, cognitively demanding dual-task condition.

AWS may have developed efficient strategies to allocate attention between formulation and monitoring, minimizing interference with memory encoding. In contrast, AWNS-SA participants, for whom the sound-avoidance task represented a novel and cognitively taxing dual-task condition, likely devoted additional working memory resources to maintain the arbitrary constraint, increasing cognitive load and disrupting referential encoding. Despite limitations in fully simulating the experience of stuttering, this comparison provides one of the first explorations of sound avoidance in AWNS, offering a potential model for understanding avoidance strategies in stuttering.

Taken together, these findings suggest that many AWS develop deeply engrained, adaptive skills in verbal avoidance. Over years of managing attentional and fluency demands, AWS may acquire efficient strategies to circumvent words or sound sequences that are likely to trigger disfluency (Newman & Ratner, 2007). Such practiced control could allow AWS to maintain perceptually fluent speech with little measurable impact on language or subsequent memory for past referents, even under substantial internal monitoring and reformulation. However, the “mental gymnastics” of such linguistic rearranging can feel cognitively taxing. From this perspective, the absence of memory differences between the AWS and AWNS groups may not indicate the absence of cognitive cost per se, but rather one aspect of the experience that can be unintentionally beneficial. These adaptive strategies may also explain why monitoring does not always impair memory: habitual control could reduce the attentional toll of monitoring, thereby preserving encoding efficiency.

### Effect of stuttering on language production and memory in AWS

At the trial level, overt stuttering was associated with reduced memory accuracy, suggesting that momentary disruptions in fluency disrupt attention to referential content. While AWS’ overall memory was comparable to AWNS, stuttered trials among AWS showed local impairments in encoding, consistent with transient shifts in cognitive focus from message formulation to motor control.

Measures of anticipation and behavioral reactions to stuttering—as measured by the *Stuttering Anticipation Scale* (SAS)—were not significantly associated with overall language or memory performance, underscoring the variability in how individuals experience and respond to stuttering anticipation (Rodgers & Jackson, 2021; Tichenor & Yaruss, 2021; Tichenor, Herring, & Yaruss, 2022). This variability highlights that the cognitive consequences of monitoring and avoidance depend on individual adaptation. Future research should further explore how individual differences in monitoring (automatic vs. strategic) differentially affect both real-time language production and long-term memory outcomes.

### Limitations

This study investigated the potential impact of linguistic monitoring on language production and subsequent memory in AWS. While AWS are assumed to frequently monitor their speech, it remains unclear how they engaged in the communication task and managed anticipated stuttering on a trial-by-trial basis. Linguistic monitoring is often covert, and AWS may attempt to circumvent stuttering moments through strategies such as word switching or circumlocution, which can prevent overt stuttering from being perceived by listeners. In our exploratory analysis, we examined how overt stuttering in a trial influenced language production and subsequent memory. However, future research is needed to fully understand how stuttering avoidance (resulting in perceptually fluent utterances) affects cognitive resources during language production and memory in AWS. This may be achieved by tailoring stimuli to AWS’ unique list of words that they anticipate stuttering on, which has been done in other studies (e.g., Jackson et al., 2022; Orpella et al., 2024).

Another potential limitation is the use of a sound avoidance task in the AWNS-SA group. While this task was designed to simulate the linguistic avoidance strategies used by AWS, it is important to acknowledge that sound avoidance was novel to AWNS-SA participants, whereas AWS has had years of experience managing stuttering-related avoidance strategies. Additionally, AWS may have engaged in an implicit dual task—simultaneously monitoring and producing language—whereas the AWNS-SA group performed an explicit dual task that required them to consciously adhere to the taboo sound rule. These differences may have imposed distinct cognitive demands across the groups. Furthermore, AWS likely employed various strategies beyond avoiding specific sounds or words to prevent overt stuttering. Individual differences in stuttering anticipation and avoidance strategies are clinically meaningful and cannot be fully captured by a single experimental task like the one imposed on AWNS-SA participants in this study.

## Conclusion

Our findings suggest that AWS can effectively coordinate monitoring, planning, and production processes without substantial disruption to attentional or cognitive resources. Although AWS tended to produce more words in the communication task, even when additional modifiers were unnecessary, suggesting that AWS may prioritize communicative clarity and social appropriateness, providing additional detail to facilitate their conversation partners’ comprehension or save face rather than to support their own memory. Additionally, we found that moments of overt stuttering while describing an object were associated with reduced memory for referents, indicating that transient disruptions in fluency can momentarily redirect attention away from conceptual encoding. However, the overall similarity in memory performance between AWS and AWNS highlights that long-term adaptation to monitoring demands may mitigate such costs. Taken together, these findings contribute to a more nuanced understanding of linguistic monitoring as a flexible control mechanism, one that can both tax and enhance processing, depending on task demands and individual adaptation. Beyond advancing psycholinguistic models of language and memory, this work underscores the importance of studying not only the nature of stuttering but also how it serves as a window into how speakers balance cognitive control, communicative goals, and social engagement in everyday conversation. Future research should further examine individual differences in how AWS linguistically navigate stuttering anticipation and the impact of these strategies on both speakers’ and listeners’ communication experiences.

## Supporting information

Yoon et al. supplementary material Yoon et al. supplementary material

## Notes

1 Participants completed a 20-minute battery of cognitive assessments measuring processing speed, working memory, and inhibitory control. The measures and results are reported in Supplemental Material 1. The cognitive battery, included as a filler task, was used to characterize participant groups and did not influence the results of the referential communication task (e.g., modification rates). Therefore, we do not discuss it further.

2 Responses from five AWS participants, including the two participants who did not complete the stuttering anticipation scale, were not recorded due to technical difficulties or experimenter errors.

3 We coded the use of filler words (e.g., um, uh) separately because filler words were frequently observed across all three groups. The results showed that the speakers in the AWNS (33.6%) and AWNS-SA (34.8%) groups produced more fillers compared to the speakers in the AWS group (23.2%).

4 We also examined a model that included Contrast Type as an additional fixed effect, but its effect was not significant. To maintain a simpler model, we report the model with three fixed effects.

5 To examine whether disfluency (e.g., filler words such as “um” or “uh”) during language production affected memory performance across groups in AWS and AWNS, we conducted an additional analysis, which revealed no significant effect of disfluency on target memory.

## Supplementary material

The supplementary material for this article can be found at https://doi.org/10.1017/S0142716425100428

## Replication package

All data and analytic code have been made publicly available at the Open Science Framework (OSF) and can be accessed at https://osf.io/xa76n.
