# Supplementary material for: Effects of stuttering and sound avoidance on reference production and memory
Source: Appl Psycholinguist. 2026 Feb 12;47:e3. doi: 10.1017/S0142716425100428 (PMC12926730; doi:10.1017/S0142716425100428)
Supplement: Yoon et al. supplementary material [file S0142716425100428sup001.docx]

**Supplemental Material 1**

**Filler Task – Cognitive Measures**

To prevent ceiling effects in memory performance (Shepard, 1967), a filler task was introduced before the recognition memory test, following prior studies on memory after conversation (e.g., Yoon et al., 2016, 2021; Saryazdi, Nuque, & Chambers, 2022). Participants completed a 20-minute battery of cognitive assessments, including measures of processing speed, working memory, and inhibitory control.

All participants completed the cognitive tasks in the same order with identical instructions.

**Processing Speed**

Processing speed was assessed with two tasks (Salthouse, 1991):

- Letter Comparison Task – Participants viewed two sets of letters and indicated whether they were identical (*S*) or different (*D*). They completed as many trials as possible in 30 seconds.
- Pattern Comparison Task – This task followed the same procedure as the letter comparison task but used visual patterns instead of letters.

**Working Memory**

Working memory was assessed using the Wechsler Memory Scale-Revised (WMS-R; Wechsler, 1987), which included:

- Digit Span (Forward and Backward) – Participants listened to a series of numbers and repeated them in the same or reverse order.
- Visual Memory Span (Forward and Backward) – Participants observed the experimenter clicking on boxes and repeated the clicking pattern.

**Inhibitory Control**

Inhibitory control was assessed using two tasks (Stoet, 2010, 2017):

- Stroop Task (Ridley, 1935) – Participants named the ink color of color words while ignoring the written word.
- Flanker Task (Eriksen & Eriksen, 1974) – Participants responded to the direction of a central arrow while ignoring distracting arrows (e.g., <<>>>).

**Group Differences in Cognitive Measures**

Descriptive statistics for the cognitive measures are shown in Table 2. Processing speed was the only measure that significantly differed between groups (*F*(2,93) = 3.39, *p* = .04). Post hoc analyses revealed that the AWNS-SA group had faster processing speed than the AWS group (*t* = 2.57, *p* = .01). However, there were no significant differences between the AWNS and AWS groups (*t* = 0.92, *p* = .36) or between the AWNS and AWNS-SA groups (*t* = -1.67, *p* = .10).

**Table S1.** Descriptive statistics (means and standard deviations) of cognitive measures across groups

|  | AWS *(n=*32) | AWNS *(n=*32) | AWNS-SA *(n=*32) |
| --- | --- | --- | --- |
| Processing Speed | 11.05 (3.78) | 11.78 (2.77) | 13.11 (3.41) |
| Working Memory | 15.97 (3.57) | 16.45 (3.27) | 17.52 (4.08) |
| Inhibitory Control 1 (Flanker) | 66.90 (67.90) | 86.03 (62.59) | 83.71 (71.71) |
| Inhibitory Control 2 (Stroop) | 88.00(72.34) | 91.97 (87.63) | 79.48 (75.72) |

*Note.* Processing speed was calculated as the mean number of correct trials from letter and pattern comparison tasks in a given time. Working memory was calculated as the mean number of correct trials from digit span and pattern span tasks. Inhibitory control (for both Flanker and Stroop tasks) was calculated as the mean reaction time difference between compatible and incompatible trials.

**Supplemental Material 2**

**Table S2.** Pre-noun modification rates: Mixed effect model with Contrast (Contrast vs. Non-contrast) and Group (Group1: AWS vs. AWNS; Group2: AWS vs. AWNS-SA) as fixed effects. The dependent measure is binary – whether the expression was pre-nominally modified or not.

|  | Estimate | SE | *z*-value | Pr(>\|z\|) |  |  | Variance | Std.Dev. |
| --- | --- | --- | --- | --- | --- | --- | --- | --- |
| *Fixed* |  |  |  |  | *Random* |  |  |  |
| (intercept) | 0.97 | 0.16 | 5.94 | <.0001 | *Subject* | (Intercept) | 0.56 | 0.75 |
| Group1 | -0.19 | 0.22 | -0.85 | 0.39 | *Item* | (Intercept) | 0.48 | 0.69 |
| **Group2** | -1.05 | 0.23 | **-4.66** | **<.0001** |  |  |  |  |
| **Contrast** | -0.58 | 0.18 | **-3.14** | **<0.01** |  |  |  |  |
| **Group1*Contrast** | -0.56 | 0.24 | **-2.30** | **0.02** |  |  |  |  |
| **Group2*Contrast** | -0.86 | 0.24 | **-3.53** | **<0.001** |  |  |  |  |

**Table S3.** Target memory: Mixed effect model with Group (Group1: AWS vs. AWNS; Group2: AWS vs. AWNS-D), Role (speaker vs. listener), and Item Type (old vs. new) as fixed effects. The dependent measure is binary – whether the response on the memory test was ‘yes’ (old) or ‘no’ (new).

|  | Estimate | SE | *z*-value | | Pr(>\|z\|) |  |  | Variance | Std.Dev. |
| --- | --- | --- | --- | --- | --- | --- | --- | --- | --- |
| *Fixed* |  |  |  | |  | *Random* |  |  |  |
| (intercept) (response bias) | 0.27 | 0.08 | 3.50 | | <.0001 | *Subject* | (Intercept) | 0.09 | 0.30 |
| (effect on response bias) |  |  |  | |  |  | Role | 0.06 | 0.24 |
| Group1 | -0.10 | 0.11 | -0.96 | | 0.34 |  |  |  |  |
| Group2 | 0.12 | 0.11 | 1.10 | | 0.27 |  |  |  |  |
| Role | 0.41 | 0.12 | 3.43 | | 0.001 |  |  |  |  |
| Group1*Role | -0.11 | 0.17 | | -0.65 | 0.52 |  |  |  |  |
| Group2*Role | 0.16 | 0.16 | | 0.98 | 0.33 |  |  |  |  |
| **Item (sensitivity)** |  |  | |  |  |  |  |  |  |
| (effect on sensitivity) |  |  | |  |  |  |  |  |  |
| **Item Type** | 3.66 | 0.11 | | **32.25** | **<.001** |  |  |  |  |
| Item*Group1 | -0.13 | 0.15 | | -0.82 | 0.41 |  |  |  |  |
| **Item*Group2** | -0.53 | 0.15 | | **-3.49** | **<.001** |  |  |  |  |
| **Item*Role** | 1.10 | 0.23 | | **4.88** | **<.001** |  |  |  |  |
| Item*Group1*Role | 0.06 | 0.31 | | 0.19 | 0.85 |  |  |  |  |
| Item*Group2*Role | 0.21 | 0.30 | | 0.69 | 0.49 |  |  |  |  |
